# Immuno-Sensing at Ultra-Low Concentration of TG2 Protein by Organic Electrochemical Transistors

**DOI:** 10.3390/bios13040448

**Published:** 2023-03-31

**Authors:** Valentina Preziosi, Mario Barra, Valeria Rachela Villella, Speranza Esposito, Pasquale D’Angelo, Simone Luigi Marasso, Matteo Cocuzza, Antonio Cassinese, Stefano Guido

**Affiliations:** 1 Department of Chemical, Materials and Production Engineering, University Federico II, P.le Tecchio 80, I-80125 Naples, Italy; 2 CNR-SPIN, c/o Department of Physics “Ettore Pancini”, P.le Tecchio, 80, I-80125 Naples, Italy; 3 CEINGE, Advanced Biotechnologies, Via Gaetano Salvatore 486, I-80145 Naples, Italy; 4 IMEM-CNR, Parco Area delle Scienze 37/A, I-43124 Parma, Italy; 5 ChiLab, Department of Applied Science and Technology, Politecnico di Torino, I-10129 Torino, Italy; 6 Department of Physics “Ettore Pancini”, University Federico II, P.le Tecchio 80, I-80125 Naples, Italy; 7 National Interuniversity Consortium for Materials Science and Technology (INSTM), I-50121 Firenze, Italy

**Keywords:** organic electrochemical transistors, functionalization, specificity, TG2 protein, diagnostic devices

## Abstract

Transglutaminase 2 (TG2) is a ubiquitously expressed member of the transglutaminase family with Ca2+-dependent protein crosslinking activity. Its subcellular localization is crucial in determining its function, and indeed, TG2 is found in the extracellular matrix, mitochondria, recycling endosomes, plasma membrane, cytosol, and nucleus because it is associated with cell growth, differentiation, and apoptosis. It is involved in several pathologies, such as celiac disease, cardiovascular, hepatic, renal, and fibrosis diseases, carrying out opposite functions of up and down regulation in the progression of the same pathology. Therefore, this fine regulation requires a very sensitive and specific method of identification of TG2, which is to be detected in very small quantities in a deregulated condition. Here, we demonstrate the possibility of detecting TG2 down to attomolar concentration by using organic electrochemical transistors driven by gold electrodes functionalized with anti-TG2 antibodies. In particular, a direct correlation between the TG2 concentration and the transistor transconductance values, as extracted from typical transfer curves, was found. Overall, our findings highlight the potentialities of this new biosensing approach for the detection of TG2 in the context of pathological diseases, offering a rapid and cost-effective alternative to traditional methods.

## 1. Introduction

Transglutaminases (TGase) are multifunctional enzymes belonging to a family of eight isozymes categorized as the blood coagulation factor XIII and TG1–7 [1]. Among them, the TG2 protein is ubiquitously distributed in almost all mammalian cell types, and it is a major player in multiple biological processes [2,3]. In addition, it is linked to several pathologies such as inflammatory diseases [4], celiac disease [5], neurodegenerative diseases [6,7], diabetes [8], tissue fibrosis [9], cancers [10,11], and cardiovascular disease (CVDs) [12,13,14] by regulating several cell functions, such as cell adhesion and extracellular matrix (ECM) formation and turnover, cell growth, differentiation, and migration, cellular fate such as cell death, survival, fibrosis, and autophagy. The essential role of TG2 in disease progression has been recently highlighted by monitoring TG2 levels in urinary samples [15] and also in chronic kidney disease (CKD) where, for patients with CKD, the TG2 concentration in urine is about 40 times larger than the values (~90 pg/mL) in healthy subjects. More in general, the involvement of TG2 in various pathological conditions makes this protein a potential therapeutic target, raising the need to monitor the related level in biological samples.

To this purpose, in the last few years, an emerging role has been played by new types of biosensors, which, as analytical devices combining a biological recognition element with a transducer to convert the recognition event into a measurable signal, represent a rapid and cost-effective method for detecting various biological molecules. Biosensors exhibit several advantages over traditional methods, including high sensitivity, rapid response time, and the ability to be miniaturized, making them suitable for point-of-care and field applications.

As far as the detection of TG2 is concerned, it has been traditionally accomplished through immunoaffinity methods exploiting optical transduction of the antigen-antibody binding events, such as the ELISA assay, which, however, can be time-consuming and requires specialized equipment. Alternative approaches have been also proposed including a streptavidin bead-based fluorometric transamidation kit or aggregation-induced emission (AIE) nanoprobe as reported in the literature [16]. Even in these cases, however, the detection of TG2 has never been assessed in the sub-femtomolar range.

On the other hand, in the rising field of biosensors, organic electrochemical transistors (OECTs) [17,18,19,20,21] are positioned as a cutting-edge class of devices thanks to their great ability to transduce and amplify the signal to be detected and to work in liquid conditions at low voltages (<1 V) with superior transconductance performances [20,22]. In its most popular architecture, the OECT can be described as a channel made of a poly (3,4-ethylenedioxythiophene) polystyrene sulfonate (PEDOT:PSS) active channel connecting the source and drain electrodes and in direct contact with the electrolytic medium. The device is completed by the gate electrode, which is also in contact with the fluid and modulates the OECT’s electrical response. When a positive voltage (V_GS_) is applied between the gate and the source, the positively charged species contained in the solution are forced to move towards the PEDOT:PSS porous ion-permeable layer, neutralizing the PSS^-^ sites and de-doping the active channel, thus decreasing the current (I_DS_) that flows between the drain and the source. These devices are classified as “depletion-mode transistors”. As a whole, this process provides a reversible way to modify the I_DS_ current, at a given drain–source voltage (V_DS_) as a function of the applied voltage between gate and source (V_GS_). In general, since the OECT operation mechanism relies on a controllable doping/de-doping process where ions can reversibly migrate in/out of the organic channel, they display unique ion-to-electron conversion properties, representing ideal tools to intimately connect biology and electronics. Indeed, in the last few years, such devices have been successfully applied for the detection of molecules involved in pathological diseases [23,24,25,26,27,28,29,30,31] and, more generally, in biomedical and biotechnological research [32,33].

In this paper, we describe the use of OECTs as biosensors for the detection of TG2 protein at ultra-low concentrations, overcoming the current limits of detection of commercial devices. To investigate device specificity, the gate functionalization process has been optimized and then assessed by an independent technique based on immunofluorescence. In addition, device sensitivity has been investigated by using three different TG2 protein concentrations, ranging from atto to picomolar, with a specific focus on the signal transconductance response. These results demonstrate the potential of biosensors to accurately and sensitively detect TG2 and suggest their applicability for the investigation and diagnosis of several pathological diseases.

## 2. Materials and Methods

### 2.1. Reagents

Primary antibodies anti-TG2 (rabbit pAbTGM2) were purchased by Novus Bio—Techne; TG2 antigen (rhTG2) was purchased by Zedira, and a secondary antibody, donkey anti-rabbit-Alexa Fluor 488, was purchased by Life Technologies.

The materials used for gate functionalization were the following: N-(3-Dimethylaminopropyl)-N′-ethylcarbodiimide hydrochloride, N-Hydroxysulfosuccinimide sodium salt (sulfo-NHS), 3-Mercaptopropionic acid (3-MPA), Ethanolamine hydrochloride, 11-Mercaptoundecanoic acid (11-MUA), Tween 20, Acetone, ethanol, isopropanol. and Phosphate-buffered saline (PBS) solution (C = 10 mM). PBS is a pH-adjusted blend of ultrapure-grade phosphate buffers and saline solutions. The PBS version used in this work contains 0.2 g/L KCl, 0.2 g/L KH_2_PO_4_, 8 g/L NaCl and 1.15 g/L Na_2_HPO_4_. No other chemical species (e.g., calcium and magnesium chloride) were included.

All these compounds were purchased by Sigma Aldrich and used without further purification. Bovine serum albumin (BSA) was purchased by Microgem.

### 2.2. OECT Fabrication

The fabrication process steps and the basic layout of the OECTs considered in this work are shown in Figure S1 and briefly described in the following, while more details have been reported elsewhere [19]. Source and drain electrodes were fabricated by e-beam evaporation (ULVAC EBX-14D) through the deposition of Ti/Au films (thickness 10  nm/100  nm) on a Si wafer (100), finished with 1 μm of thermal SiO_2_, and by subsequent standard photolithographic steps. In particular, the gold etching for the definition of the source/drain electrodes was performed using the ready-to-use solution TechniEtch™ACI2 (from Microchemicals GmbH), for 30s at RT.

For the transistor channel, a solution of PEDOT:PSS layer (Clevios PH 1000), doped with 5 vol% of ethylene glycol, 0.1 vol% of dodecyl benzene sulfonic acid, and 1 wt% of GOPS (3-glycidyloxypropyl)trimethoxysilane) was spin coated (with a final thickness equal to 200 nm) on the source and drain electrodes. The PEDOT:PSS active area was patterned using photolithography (Microchemicals AZ9260 resist) and O_2_ plasma etching. In this way, a width/length ratio (W/L) of 30 (6 mm/0.2 mm) for the active channel was achieved. The devices were completed by irreversibly bonding polydimethylsiloxane (PDMS) chambers (with an internal volume of 150 μL) on the SiO_2_ surface, after a careful alignment with the PEDOT: PSS channel layout.

#### 2.2.1. Gate Functionalization

To allow the selective and sensitive detection of TG2, gold wires working as gate electrodes were bio-functionalized with an anti-TG2 protein. Specifically, gate biofunctionalization consists of binding an organic molecule, in this case the anti-TG2 antibody, to the electrode surface and exploiting the antibody-antigen reaction to detect the presence of specific molecules in solution.

**Figure 1:**
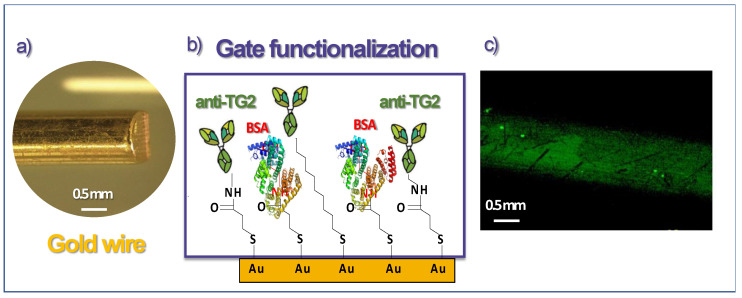
(**a**) Image of the gold wire used as gate electrode; (**b**) Cartoon of gate functionalization; (**c**) CLSM imaging of the gate electrode.

The protocol consists of the following steps: first of all, the gold wires (purity: 99.99%, purchased by Franco Corradi sas) with a diameter of 1 mm (Figure 1a) were properly cleaned in acetone and isopropanol for 10 min each in an ultrasonic bath. Once cleaned, they were immersed in a pure ethanol solution containing 10 mM thiols (3-MPA and 11-MUA in 10: 1 molar ratio) for 24 h, after which they were washed several times in ethanol and deionized water and dried under air flow. This step allows the formation of a layer on the gold surface known as SAM (self-assembled monolayer) [34,35], with a carboxylic group (COOH) at one end and a thiol group (SH) at the other end, the latter being able to bind covalently to gold [36]. Then, the gold wires were immersed in an aqueous solution containing 1-Ethyl-3-(3-dimethylaminopropyl)carbodiimide (EDC) at 200 mM and N-hydroxysulfosuccinimide (sulfo-NHS) at 50 mM for 2 h at 25 °C, after which they were washed with High Performance Liquid Chromatography (HPLC) water, which is an ultrapure water with low level of ions and less impurities than deionized water. This reaction produces the activation of the terminal carboxyl groups of each thiol through the formation of amide bonds between the aforementioned COOH groups and the amino functions of the EDC. Such a step allows the formation of hydrogen bonds inside the thiol layer, with the consequent achievement of a dense electrostatic network; the system thus formed is called chem-SAM. The gold electrodes were then immersed in 100 μL of 10 mM PBS at pH = 7.4 containing the antibodies of interest (100 μg/mL of anti-TG2) for one hour, after which they were first rinsed with PBS solution containing 0.1% Tween 20, a detergent commonly used to improve the removing action of any impurities, and finally with PBS only. The antibody functionalization of the gate electrodes makes the final device selective towards a very specific target molecule (in our case, TG2) [37,38]. Subsequently, the gates were immersed in a 1 M solution of ethanolamine in 10 mM PBS for 1 h at 25 °C, then washed with deionized water. The addition of ethanolamine to the system serves to block the activated carboxyl groups not bound to any antibody. Finally, the gold wires were immersed in a 1.5 μM solution of bovine serum albumin (BSA) in 10 mM PBS for 1 h at 25 °C in order to further minimize any non-specific bond; it has been shown in previous studies, indeed, that the presence of BSA increases the quality of detection by significantly lowering the so-called Limit of Detection [27]. The system thus formed, placed immediately above the chem-SAM, takes the name bio-SAM (Figure 1b).

Before using the OECT for sensing measurements and to demonstrate the proper gate functionalization, fluorescence imaging was performed to verify the presence of anti-TG2 antibodies on the gold surface. To this purpose, the biofunctionalization process was modified with an additional step: after immersing the gold gate in the anti-TG2 antibody solution, it was immersed in a solution of PBS containing fluorescent secondary antibodies (anti-rabbit-488 conjugated, dilution 1:200) for 1 h at room temperature. As the secondary antibodies bind specifically to the primary ones, adhesion of the primary antibodies on the gate surface using a confocal laser scanning microscopy (CLSM—Zeiss LSM5 Pascal) could be verified. After that, the functionalized gate was rinsed with PBS and dried under air flow. CLSM imaging during the various functionalization process steps was carried out, and the related images are reported in Figure 1c and in Figure S2 in the Supplementary Materials.

#### 2.2.2. OECT Measurements

OECT devices were electrically tested by using a probe station (supplied by EverBeing) connected to a two-channel source meter (Keithley 2602B) and controlled by Labview software. The OECT’s electrical response was carefully assessed by performing two main types of measurements.

In a first set of experiments, the I_DS_ current and the corresponding I_GATE_ were measured over time by applying a fixed V_DS_ = −0.1 V and V_GS_ pulses with variable amplitude from 0 to 0.8 V (rise time = 100 ms). V_GS_ pulses, with a duration of 50 s and a step ∆V_GS_ = 0.1 V, were progressively applied in order to allow the I_DS_ current to reach the steady state condition. Before and after any pulse, V_GS_ was set to zero in order to recover the initial I_DS_ value. In a second run of measurements, the devices were tested in terms of transfer curves. In this case, while keeping V_DS_ value fixed at −0.1 V, V_GS_ was swept from −0.1 to 0.8 V with a step ∆V_GS_ = 10 mV. In these experiments, a delay time of 5 s was imposed between the V_GS_ application and the corresponding I_DS_ recording. Before starting any biosensing experiment, the involved OECT channels were immersed in bi-distilled water for at least 2 h. Then, pulse or transfer curve measurements were performed using gate electrodes before and after the various incubation steps in PBS solutions containing different concentrations of rhTG2 protein. PBS 10 mM was invariably utilized as the electrolyte for the OECT tests. In all experiments, the immersed gate area was fixed at 8 mm^2^.

## 3. Results

**Figure 2:**
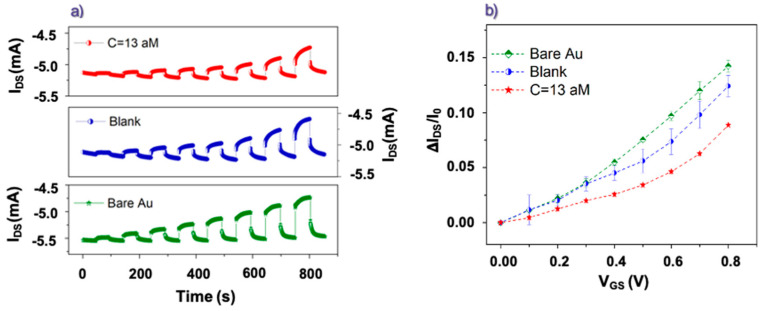
(**a**) IDS vs. time (pulse) curves recorded in PBS using a pristine gate electrode (Bare Au), a gate electrode functionalized with anti-TG2 antibodies before any incubation step (Blank) and after the incubation in solution with attomolar concentration of rhTG2 protein (C = 13 aM). (**b**) Corresponding average values of the I_DS_ modulation extracted as a function of V_GS_. Error bars represent the standard deviations.

Figure 2a reports some pulse measurements achieved while OECTs were driven by differently functionalized gate electrodes. Here, V_GS_ was increased from 0 V to 0.8 V (a step of 0.1 V), while V_DS_ was kept constant at −0.1 V. In this case, the response obtained with a pristine (bare) gate Au electrode was compared with those recorded using the same electrode, once functionalized with anti-TG2 antibody, before (blank) and after the incubation in solution with an attomolar (13 aM) concentration of rhTG2 protein. All the curves in Figure 2a confirm clearly the proper operation of the considered PEDOT:PSS-based OECT as depletion-mode transistors [39] since a decrease (in absolute value) of the I_DS_ current upon the application of positive V_GS_ voltages can be observed. As aforementioned, this occurrence is related to the electronic de-doping action carried out by the cationic species (namely, Na^+^ and K^+^ given the use of PBS as an electrolyte) penetrating in the PEDOT: PSS channel.

The substantial reversibility of this phenomenon is confirmed by the tendency of I_DS_ to recover the initial value when V_GS_ is brought back to 0 V. Figure S3a shows the gate (I_GATE_) current curves over time recorded in parallel with the corresponding I_DS_ ones in Figure 2a. The I_GATE_ currents exhibit the characteristic behavior of large peaks occurring upon the V_GS_ switching and steady state values (at the end of the V_GS_ pulses) that are several orders of magnitude lower than the I_DS_ values (see Figure S3b). These features are basically related to the capacitive behavior of the polarizable gold gate electrodes [40].

From the pulse tests shown in Figure 2a, the I_DS_ current modulation values (ΔI_DS_/I_0_), as a function of V_GS_, have been extracted using the basic expression ΔI_DS_/I_0_ = (I_I_ − I_0_)/I_0_, where I_0_ and I_I_ are, respectively, the I_DS_ values measured just before the start and just before the end of any V_GS_ pulse [41]. The final ΔI_DS_/I_0_ modulation values shown in Figure 2b exhibit a maximum value of about 15% obtained at V_GS_ = 0.8 V. Moreover, the data in Figure 2b demonstrate clear differences in the OECT current modulation values as a function of the specific gate functionalization. The ΔI_DS_/I_0_ values achieved by the gate electrodes functionalized with anti-TG2 antibodies after the incubation in the PBS solution with rhTG2-protein attomolar concentration are indeed smaller, throughout the analyzed V_GS_ range, than those estimated for the same functionalized electrodes before the incubation step. On its turn, the functionalization step with the anti-TG2 antibody reduces the gating capability as compared to the pristine (bare) Au electrode (this effect is more evident for V_GS_ larger than 0.4 V). As a whole, these findings confirm that the device operation is able to recognize the presence of the antibodies bonded to the gate electrode surface and, above all, the presence of antigens as a consequence of the incubation step in a proper solution. It should be stressed that the ability of the device to detect the presence of rhTG2-protein in saline solutions at the attomolar level (i.e., 13 aM corresponds to about 1 fg/mL) is a very significant result, going beyond the sensitivity of the presently available enzyme-linked immunosorbent assay detection (ELISA) tests which work effectively down to tens of pg/mL. Even more recent innovative electrochemical approaches devoted to improving the diagnosis of hepatocellular carcinoma (HCC) are able to detect minimum TG2 concentrations, being however larger than some hundreds of fg/ml [42].

Figure 2b also allows outlining the specific dependence of ΔI_DS_/I_0_ values on V_GS_. For the data recorded with the gate incubated in the PBS solution containing the rhTG2-protein, this feature is particularly clear in the low V_GS_ region between 0 and 0.4 V. This observation prompted us to investigate more carefully the OECT response in terms of the transfer curves, focusing our attention, in particular, on the transconductance (g_m_) values defined as the derivative of I_DS_ as a function of V_GS_ (i.e., g_m_ = δI_DS_/δV_GS_).

In the transistor theory, g_m_ is a fundamental parameter that reflects the signal amplification performances of the device. Given the volumetric effect involving the entire active channel bulk during the OECT operation, these devices have been demonstrated to display very large g_m_ values when compared to other transistor families [22].

**Figure 3:**
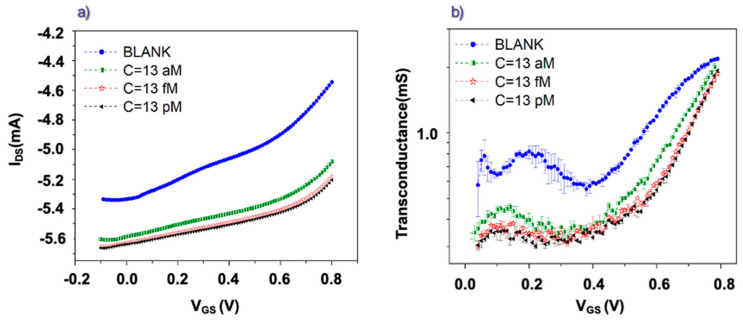
(**a**) OECT transfer curves recorded (at V_DS_ = −0.1 V) for gate electrodes functionalized with anti TG2 antibodies before (Blank) and after incubation in solutions with different concentrations of rhTG2 protein. (**b**) Transconductance values extracted from the transfer curves in the panel (**a**).

Figure 3a reports transfer curves measured using gate electrodes functionalized with anti-TG2 antibodies before and after the incubation in PBS solutions containing different concentrations (from atto- to picomolar) of rhTG2 protein. The time delay between the application of any V_GS_ value (with a step of 10 mV) and the acquisition of the corresponding I_DS_ current was set to 5 s to favor the achievement of the same steady state conditions as the protocol based on the pulse measurements. The curves in Figure 3a exhibit a progressive increase (in absolute value) of the I_DS_ baseline (i.e., the I_DS_ current measured at negative V_GS_). However, this effect was not observed to the same extent throughout the various experiments and, in our opinion, should be associated more likely to underlying ion diffusion processes slightly modifying the active channel conductivity over time. More interestingly, the transfer curves recorded after the different incubation steps were characterized also by a progressive change in their slope. This feature is also shown in Figure 3b, where the corresponding transconductance (g_m_ = δI_DS_/δV_GS_) values are reported. All the curves in Figure 3b display a mainly constant behavior in the low V_GS_ region (mainly between 0 and 0.3 V) coherently with the linear regime of the transistor operation [40]. Conversely, for larger V_GS_ values (>0.4 V), the linear response is lost, and g_m_ tends to rapidly increase with V_GS_. In general, this steeper trend cannot be described in the framework of the widely discussed OECT model developed by Bernards and Malliaras starting from the transistor theory [40]. Tentatively, this occurrence could be ascribed to the initial emergence of water electrolysis effects or, alternatively, to an intrinsic non-linear electrical behavior of the device suggesting that the charge de-doping effect (i.e., the I_DS_ decrease) associated with the cation penetration in the PEDOT:PSS active channel becomes progressively more and more effective at increasing V_GS_.

**Figure 4:**
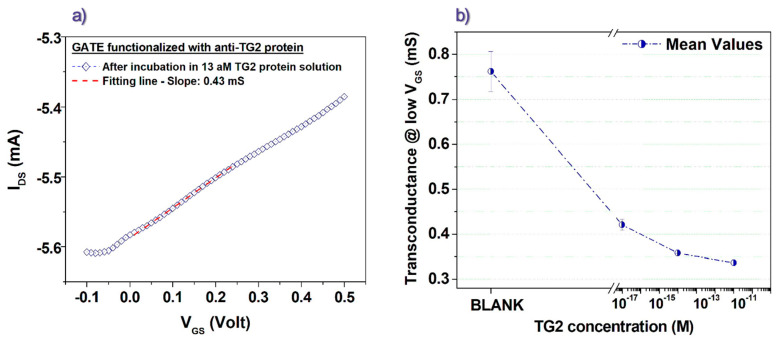
(**a**) Linear fitting in the low V_GS_ region of a transfer curve (at V_DS_ = −0.1 V) obtained for a gate electrode functionalized with anti-TG2 antibodies after incubation in solution with attomolar concentration of rhTG2 protein. (**b**) Transconductance values extracted as a function of the rhTG2 protein concentration in the incubation solution.

While focusing again on the low V_GS_ voltages (between 0 and 0.3 V), we infer the presence of a correlation between the g_m_ values and the concentration of the rhTG2 protein associated with the corresponding incubation step. Indeed, it can be observed that the transconductance progressively decreases as the concentration of the rhTG2 protein increases. To get a more precise quantitative estimation of the g_m_ values in the low V_GS_ region, we performed simple linear fits, as shown in Figure 4a, for a transfer curve recorded after the incubation step in the rhTG2 attomolar concentration. Other examples of the fitting procedure are reported in Figure S4 of the Supplementary File. Following this way, we achieved the plot in Figure 4b, which clearly shows that the g_m_ values estimated in the low V_GS_ region can be associated with the rhTG2 protein concentration used in the corresponding incubation step. In particular, for the experiment involving the rhTG2 attomolar (13 aM) concentration, a decrease larger than 40% of the g_m_ value was produced by the incubation step. When the femtomolar concentration was analyzed, on the other hand, a g_m_ reduction of about 52% in comparison to the related Blank experiment was observed. Finally, while further increasing the TG2 concentration, the progressive percent variation of g_m_ tends to saturate (being about 55% for the picomolar concentration) coherently with a less pronounced modification of the overall transfer curve. This saturating trend of the detection parameter at increasing antigen concentrations resembles the experimental data discussed in previous studies, which have focused on similar sensing schemes where organic transistors, driven by functionalized gate electrodes, were applied to the ultra-sensitive detection of biomolecules [27]. This finding should be basically related to a progressively lower impact of the antigen-antibody binding effect on the electrical response when the antigen concentration overcomes a threshold, which should depend on the specific target molecule but also on other experimental details [43,44].

## 4. Discussion and Conclusions

As a whole, the experimental findings here discussed agree well with many reports from the literature demonstrating the enhanced sensitivity performances achievable by exploiting the response of organic (semi)conducting channels driven by properly functionalized gate electrodes. The observed reduced modulation of the I_DS_ current upon the occurrence of the antibody-antigen reaction at the gate electrode surface, in particular, is a commonly observed feature related to the associated depressed gating efficiency [45].

In any case, it is important to mention that the sensitivity performances of this biosensing approach are affected not only by the adopted biofunctionalization strategy but also by the specific response characteristics of the employed devices. In our study, in particular, given the gold area (~8 mm^2^) immersed in the electrolyte (i.e., PBS) during the OECT tests and considering the volumetric capacitance (C_V_~40 F·cm^−^^3^) associated with the whole PEDOT-PSS/electrolyte interface, the capacitance (C_G_) between the gate and electrolyte is much lower than C_V_. This feature means that most of the applied external V_GS_ voltage drops at the gate/electrolyte interface, thus explaining the observed reduced capability to modulate the I_DS_ current (never exceeding 15% for V_GS_ values up to 0.8 V). For this reason, the considered PEDOT:PSS OECTs operate in the so-called “limited gating regime”. On the other hand, in this specific operation mode, the OECT response is also more sensitive to the different functionalization of the gate electrode since the bio-recognition events occurring at gate/electrolyte interfaces have a direct impact on the associated V_GS_ drop during the device operation [39]. More specifically, the gate functionalization and the related antigen-antibody binding affect directly the C_G_ capacitance, and given the aforementioned gating conditions, the I_DS_ current variation gets basically determined by the change of this parameter. In the framework of the basic OECT model [39,40], derived from the transistor theory and providing satisfactory predictions under certain assumptions, indeed, the “limited gating regime” provides a direct proportionality between the transconductance g_m_ and the C_G_ value (g_m_ ∝ (C_G_∗C_V_/C_G_ + C_V_)~C_G_). This condition should explain the observed increased sensitivity of this last parameter to the antigen concentration.

In conclusion, the remarkable detection performances here discussed are very encouraging for the development of innovative biosensing devices based on OECT. In particular, future work will be aimed at optimizing the introduced experimental approach for the detection of TG2 in serum samples and the comparison between CKD patients and healthy subjects. However, the adopted scheme could be easily extended for the detection of other proteins involved in pathological conditions especially when very low molecule concentration (down to attomolar) can be useful for the early diagnosis and management of various diseases.

## Data Availability

Data is contained within the article or Supplementary Materials.

## Supplementary Materials

The following supporting information can be downloaded at: https://www.mdpi.com/article/10.3390/bios13040448/s1, Figure S1: Experimental setup; (a) Fabrication steps; (b) anti-TG2 protein-antigen bound; (c) OECT layout.; Figure S2: CLSM imaging of the gate electrode during functionalization process: from left to right: before incubation in secondary anti-TG2; after incubation in anti-TG2 PBS solution; at the end of functionalization process; after one night. Figure S3: (a) I_GATE_ vs. time (pulse) curves corresponding to the I_DS_ measurements shown in Figure 2a of the main text. (b) Average I_GATE_ values as a function of V_GS_. Error bars represent the standard deviations. Figure S4: Linear fitting in the low V_GS_ region of transfer curves (at V_DS_ = −0.1 V) obtained for a gate electrode functionalized with anti-TG2 antibody (a) before any incubation step (Blank), (b) after incubation in a TG2 protein solution with femtomolar concentration, (c) after incubation in a TG2 protein solution with picomolar concentration.
