# Supplementary material for: Immuno-Sensing at Ultra-Low Concentration of TG2 Protein by Organic Electrochemical Transistors
Source: Biosensors (Basel). 2023 Mar 31;13(4):448. doi: 10.3390/bios13040448 (PMC10136445; doi:10.3390/bios13040448)
Supplement: Supplementary file 1 [file biosensors-13-00448-s001.zip › biosensors-2251941-supplementary.pdf]

biosensors

Supplementary Materials

Immuno-Sensing at Ultra-Low Concentration of TG2 Protein by Organic Electrochemical Transistors

Valentina Preziosi [1,*], Mario Barra [2,*], Valeria Rachela Villella [1,3], Speranza Esposito [1,3], Pasquale D'Angelo [4], Simone Luigi Marasso [4,5], Matteo Cocuzza [4,5], Antonio Cassinese [2,6] and Stefano Guido [1,3,7]

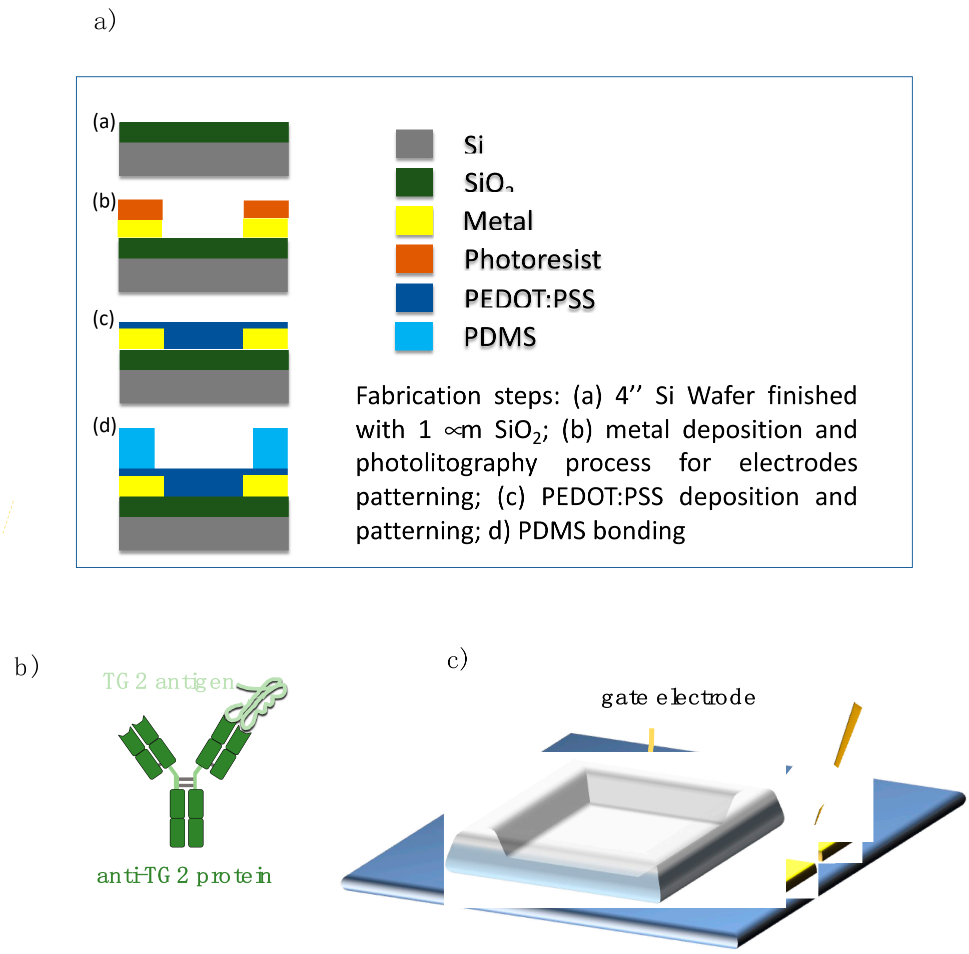

Figure S1. Experimental setup; (**a**) Fabrication steps; (**b**) anti-TG2 protein-antigen bound; (**c**) OECT layout.

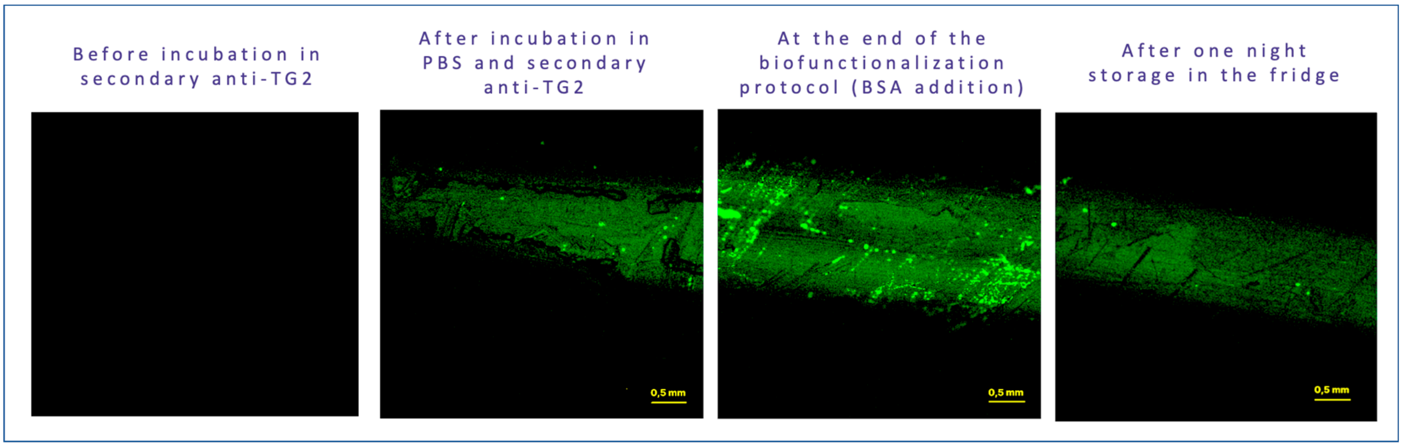

Figure S2. CLSM imaging of the gate electrode during functionalization process: from left to right: before incubation in secondary anti-TG2; after incubation in anti-TG2 PBS solution; at the end of functionalization process; after one night.

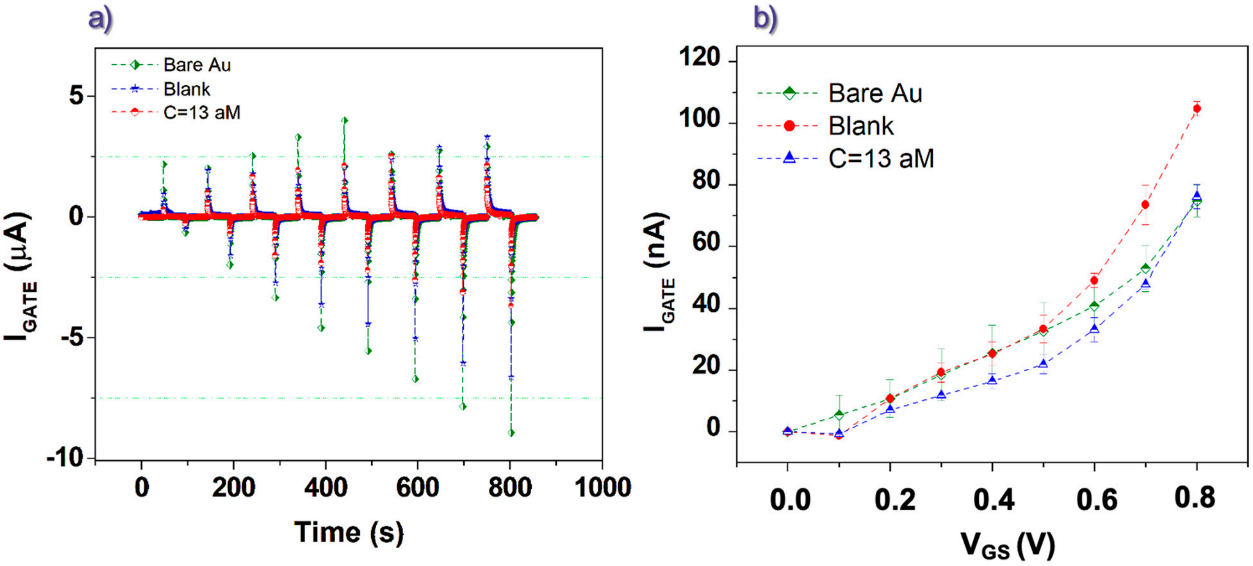

Figure S3. (**a**) I_{GATE} vs. time (pulse) curves corresponding to the I_{DS} measurements shown in Figure 2a of the main text. (**b**) Average I_{GATE} values as a function of V_{GS}. Error bars represent the standard deviations.

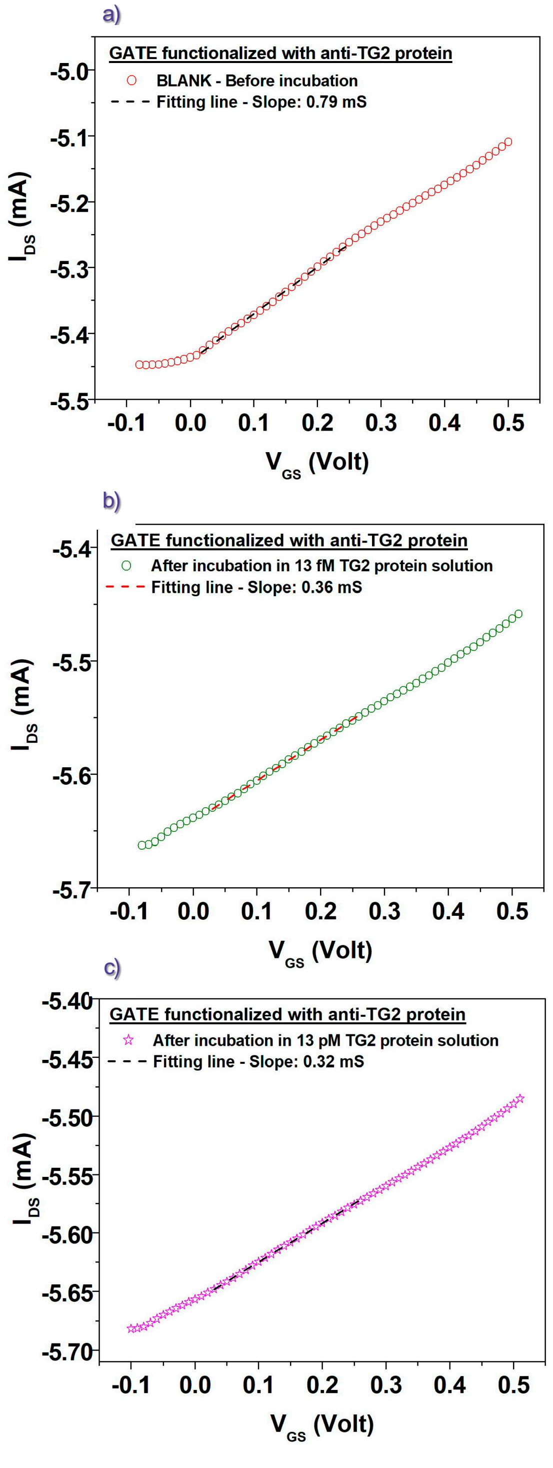

Figure S4. Linear fitting in the low V_{GS} region of transfer curves (at $V_{DS} = -0.1$ V) obtained for a gate electrode functionalized with anti-TG2 antibody (**a**) before any incubation step (Blank), (**b**) after incubation in a TG2 protein solution with femtomolar concentration, (**c**) after incubation in a TG2 protein solution with picomolar concentration.
